# Balancing long-term and short-term strategies in a sustainability game

**DOI:** 10.1016/j.isci.2024.110020

**Published:** 2024-05-27

**Authors:** Francesco Bertolotti, Sabin Roman

**Affiliations:** 1 School of Industrial Engineering, LIUC Università Cattaneo, Castellanza, Province of Varese, Italy; 2 Centre for Study of Existential Risk, University of Cambridge, Cambridge, UK

**Keywords:** Applied computing, Energy Modelling, Social sciences, Economics

## Abstract

Our society is marked by a tension between short-term objectives, such as economic growth, and long-term sustainability goals, including mitigating resource depletion. In such a competitive setting, it is crucial to ascertain whether a system can maintain long-term viability and, if so, how. This article aims to enhance the understanding of this issue by analyzing how sustainability concerns change over time by means of a game, and the effect of this variation on the final status of a system. Leveraging insights from the game, we implement an agent-based model to elicit the tension between short-term objectives and sustainability, emphasizing the influence of individual actions on the overall system. The simulation results suggest that the likelihood of a collapse is contingent upon the availability of resources and the manner in which information regarding these resources is gathered and utilized. Finally, the paper proposes practical suggestions for managing this kind of system.

## Highlights

• Game where short-term political threats oppose long-term sustainability challenges
• Game encoded into an agent-based model, allowing players to interact on a network
• Results suggest collapse could happen with inability to process long-term information

## Graphical abstract

**Figure:**
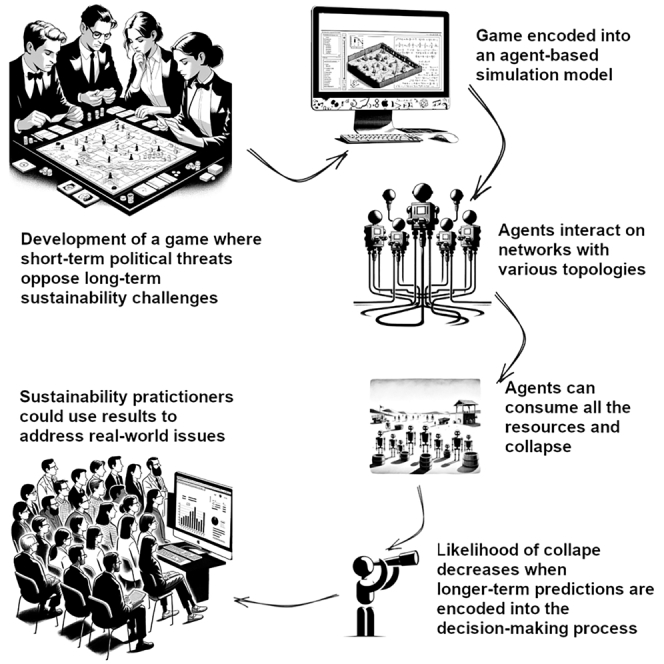

## Introduction

The issue of sustainability has flourished into a diverse set of scientific, economic, and technological developments. While historically there have been concerns about sustainability, these have mostly been limited to specific regions, such as in the case of the Republic of Venice which faced challenges with depleting wood stocks, critical for shipbuilding, due to deforestation.^1^ However, the modern sustainability movement was born with the publication of Silent Spring,^2^ which raised concerns regarding pesticide use and its environmental effects. Limits to Growth^3^ followed a decade later, and presented the first global integrated and systemic model, showing how the current development pathway of industrial society was not sustainable over the next century. The Brundtland Report^4^ established guidelines for sustainable development and informed the first United Nations conference on the topic. In recent decades, sustainability has been framed in terms of planetary boundaries^5^ and computational modeling has regularly employed in field through global integrated models.^6^

In the present work, we aim to contribute to the sustainability literature by exploring a model of a game played by interacting agents, wherein each can opt between an industrial (fossil-fuel based) path or an ecologically sustainable one, along with the possibility of initiating warfare to gain an advantage. This work is not meant to be a high fidelity reproduction of real world dynamics, but only to capture certain intuitions about how the dynamics can unfold in a simplified setting and check their outcomes. Due to the complexity of the underlying real systems, even a simplified description implies a dynamical evolution that is not obvious and non-trivial.^7^^,^^8^ If the implications of the model were straightforward, there would be little need for computer simulation. However, this does not appear to be the case, and our effort is an exercise in elucidating the implications of the assumptions, providing both a research exercise on how to map ideas to their logical outcomes and an educational tool for understanding how the dynamics and limitations of a serious sustainability game (SSG). Furthermore, given the large number of SSGs we noticed that a systematic computational analysis of the games was lacking. The present work can serve as a blueprint for evaluating other games and their implications.

We aim at contributing to sustainability literature in a 3-fold way: (1) discovering core dynamics and uncertainties when investing in renewable, non-renewable, or military sectors, (2) showing the potential outcomes of the competitive game, and (3) demonstrating tradeoffs between long-term collective and short-term individual strategies and suggest ways to gain efficiencies overall, where the term “collective” defines a scenario wherein agents refrain from depleting the shared resource pool, while concurrently, no single agent possesses the capability to monopolize or exhaust these resources entirely. So, the contribution strives to provide foundations for future empirically grounded researches generating new questions and exploring unknown dynamics.

The simulation model embodies as a game between different players. Thus, it lies at the intersection of agent based computing and of SSGs, which have emerged as a powerful tool in instructing the public, stakeholders and decision-makers regarding economic and environmental challenges in complex and uncertain socio-ecological system when a collapse risk is present.^9^ The game is designed to culminate in one of three possible conclusions: collapse, victory for one player, or collective survival. Results illustrate the impact of environmental conditions—specifically, the quantity of available resources—and players’ information processing on the determination of the final scenario.

In the next section we highlight the recent literature relevant to our topic, namely agent-based modeling (ABM) employed in sustainability research and the use of open world, multi-agent, computer-based SSG. The Methods section outlines the game played, along with a formal specification. While the rules of the game are conceptually simple and could in principle be played as board game, the precise specification and analysis reveals a great deal of complexity even in an apparently naive setting. This work builds upon and extends a prior publication,^10^ which focused on the evolution of risk preferences.

In the present paper we generalize the game and place the agents on a network that determines who they interact with. Subsequently, the Method section details the implementation of the game into an agent-based model, particularly focusing on agents’ decision-making processes, and the experimental setting employed to run the model and generate the results. The Results section presents the analysis of the agent-based model exploration, and shows the emerging final distribution of players, namely if there’s one winner, a collapse or multiple winners. Finally, we outline the limitation of the study and provide conclusions for future work.

### Literature review

There is ample literature documenting the urgent sustainability problems facing the modern world.^11^ One response to these pressing issues has been to develop SSGs as more engaging educational and instructional tools. SSGs formalize key aspects of ecological and instructional dynamics as well as their constraints. Likewise, they provide valuable teaching on how to manage and cope with ever-increasing sustainability challenges.^12^^,^^13^ The last decades have seen substantial growth in the literature on SSGs, with games varying from board games, to computer and web-assisted gaming, to role-playing or a mix of these elements.^14^ Table 1 highlights some examples of open-world, computer-based serious games developed to facilitate sustainable goals.^15^ The topics covered vary from agricultural land usage, to energy conservation, to urban development, and provide a way to mimic real world dynamics in a controlled setting, giving practice and insight to facilitate policy making in the real world. The examples in Table 1 were chosen to reflect the methodology (computer assisted) and focus (agriculture, energy) of the present study.

**Table 1:** List of open-world or simulation games for sustainable development (SD) goals

Type	Examples	Aims and themes
Online sandbox game	3rd World Farmer Climate Challenge Stop Disasters! Energyville Encon City	Sustainability of agricultural land use Renewable energy sources and politics Natural disasters prevention Sustainable energy supply Energy conservation
Computer simulation game	New Shores: A Game for Democracy World Climate Green City Tragedy of the Tuna The UVA Bay Game	Green project management Global warming decision-making Green urban development Water resources management Sustainable products and services

Furthermore, there are also game theoretical treatments of sustainability,^16^ with work ranging from developing cooperative frameworks,^17^^,^^18^ analyzing supply chains,^19^^,^^20^ addressing stakeholder decision-making and corporate accountability^21^^,^^22^^,^^23^^,^^24^ or employing evolutionary game theory to understand sustainable energy development.^25^ Our work can be considered to be in a similar spirit to the game theoretic contributions, aiming to improve strategic decision-making, however, we perform our analysis in a broader ABM framework.

In this paper, we propose a sustainability game that involves investments in either renewable, non-renewable, or military sectors. The game can be mapped to a discrete dynamical system that allows us to explore its outcomes in a systematic way.^26^ Dynamical systems have a long tradition of being applied in sustainability settings starting with the pioneering work of Jay Forrester,^27^ the Limit to Growth report^3^ and recent literature on societal collapse.^28^^,^^29^^,^^30^^,^^31^^,^^32^^,^^33^^,^^34^ However, since the game is played between agents, this makes the overall framework fall also within ABM. More recently, both agent-based and system dynamics models have been built to face sustainability issues,^35^^,^^36^ especially to address the complexity related to Table 2.^37^

**Table 2:** Behavioral variables and parameters for agent *i*

Name	Description	Allowed values
$pb_{1}^{i}$	Preference to produce *k* using *k*	$pb_{1}^{i}\in \left[0,1\right]$
$pb_{2}^{i}$	Preference to produce *k* using *g*	$pb_{2}^{i}\in \left[0,1\right]$
$pb_{3}^{i}$	Preference to produce *g* using *k*	$pb_{3}^{i}\in \left[0,1\right]$
$pb_{4}^{i}$	Preference to produce *g* using *g*	$pb_{4}^{i}\in \left[0,1\right]$
$pb_{5}^{i}$	Preference to produce *r* using *k*	$pb_{5}^{i}\in \left[0,1\right]$
$pb_{6}^{i}$	Preference to produce *r* using *g*	$pb_{6}^{i}\in \left[0,1\right]$
$pb_{7}^{i}$	Preference to produce *b* using *g*	$pb_{7}^{i}\in \left[0,1\right]$
$pa_{1}^{i}$	Preference to produce a player with *k*	$pa_{1}^{i}\in \left[0,1\right]$
$pa_{2}^{i}$	Preference to produce a player with *g*	$pa_{2}^{i}\in \left[0,1\right]$
$pa_{3}^{i}$	Preference to produce a player with *r*	$pa_{3}^{i}\in \left[0,1\right]$
$rs_{r}^{i}$	Risk sensitivity regarding resource production	$rs_{r}^{i}\in \left[-1,1\right]$
$rs_{a}^{i}$	Risk sensitivity regarding attack	$rs_{a}^{i}\in \left[-1,1\right]$

ABM is a computational approach that has gained significant popularity and utility in the field of sustainability^38^ and socio-ecological systems.^39^ It offers a powerful framework for simulating complex systems, understanding emergent behavior, and exploring various scenarios to inform decision-making.^40^ Sustainability issues often involve spatial dynamics, such as urban development, land-use change, or ecosystem fragmentation.^41^ ABM can incorporate geographical information and spatial interactions, making it suitable for modeling spatially explicit sustainability problems. Early agent based models, such as Sugarscape,^42^ have been used to illustrate how large scale features in stochastic socio-ecological systems can emerge, provide a toolkit for understanding the phenomena. In particular, ABMs have been employed to understand past cases of societal collapse, such as the Anasazi,^43^ the Maya,^44^ or the Bronze Age Collapse.^45^ Applications of agent based models have seen continued growth in tackling sustainability issues and present a way to guide policy intervention.^46^ Also, it has been used to investigate sustainable behavior adaptation.^47^

ABMs have seen extensive use in geographical systems,^48^^,^^49^^,^^50^ ecological economics,^51^ markets management,^52^ urban planning,^53^^,^^54^ energy management,^55^^,^^56^ policy making,^57^ and in conceptualizing ideas of resilience and sustainability.^58^ More specific applications include climate change adaptation,^59^^,^^60^^,^^61^ impacts on agriculture,^62^^,^^63^^,^^64^ the transition to electric vehicles,^65^^,^^66^^,^^67^ and sustainable tourism.^68^ Given the wide variety of systems at play, ABM often requires interdisciplinary collaboration between scientists, policymakers, and stakeholders to ensure the models adequately capture the complexity of sustainability challenges and their implications.^69^^,^^70^ The game we propose follows in both the tradition of modeling large scale societal dynamics, with the possibility of socio-ecological collapse,^71^^,^^72^ but also the prospect of transitioning to a future wherein multiple players co-exist sustainably.

### Methods

This section outlines the methodology, organized into four subsections: the sustainability game framework, detailing the game’s rule in both natural and formal language; the ABM, where the sustainability game framework is embodied into a simulation model; the model assumptions, where the ratio underlying the modeling decisions regarding the implementation of the game into a simulation model is explained; and the experimental design strategy, wherein the techniques used to obtain results from the simulation model are detailed.

#### Sustainability game

The purpose of this game is to create a trade-off between collective long-term sustainability and individual short-term production and military goals in a competitive environment. In this setting, agents can achieve short-term benefits by acting unsustainably. This section presents the game in two ways: first, it provides a qualitative description, and then it offers a mathematical formalization of the model. The goal of this 2-fold representation is to ensure both explainability and replicability for the paper.

##### Qualitative description

The main component of the game consists of four stocks of different types of blocks, each standing for a set of real-world resources. For clarity, each block is assigned a color: brown, green, red, and black

(1) the biosphere is modeled by brown blocks;
(2) the sustainable industrial capacity is modeled by green blocks;
(3) the non-sustainable industrial capacity is modeled by black blocks.
(4) the military capacity is modeled by red blocks; The game proceeds in turns. Initially, a predetermined final turn is set. Once this final turn is reached, the game ends. In each turn, a player decides on the production of new blocks and whether to attack another player. Every player begins with an identical initial provision of green, red, and black blocks. In contrast, the brown blocks, representing a stock of natural resources, are not owned by any individual player, embodying a shared resource. The game concludes for a player when any of the following conditions are met: the player remains the sole contender, having defeated others through multiple aggregations; the shared stock of brown blocks reaches zero, signifying the complete consumption of the biosphere and the consequent demise of all players; the player no longer possesses any green or black blocks, indicating they were the target of a successful aggression; or the game reaches its final turn, and all remaining players are declared winners. Summing up, a player can achieve victory either individually or collectively, and specularly, defeat can be either individual or collective. Thus, the potential outcomes introduce a tension between individual short-term and collective long-term objectives.

**Figure 1:**
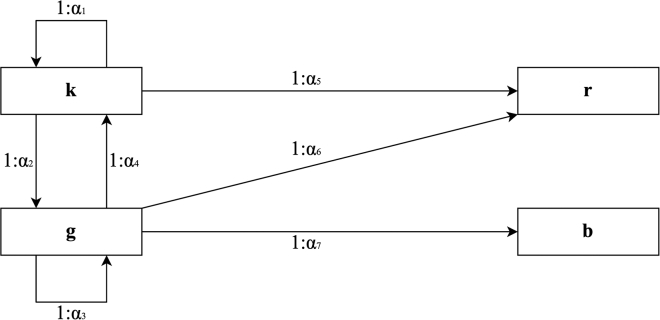
Scheme of production rules

The blocks representing sustainable and non-sustainable industrial capability (respectively, green and black), can be employed to generate new blocks every turn, according to the following rules (graphically presented in Figure 1).

(1) From 1 black block, $\alpha_{1}$ black blocks can be generated;
(2) from 1 black block, $\alpha_{2}$ green blocks can be generated, destroying the black blocks employed in the transformation;
(3) from 1 green block, $\alpha_{3}$ green block can be generated;
(4) from 1 green block, $\alpha_{4}$ black block can be generated, destroying the green blocks employed in the transformation;
(5) from 1 black block, $\alpha_{5}$ red block can be generated;
(6) from 1 green block, $\alpha_{6}$ red block can be generated;
(7) from 1 green block, $\alpha_{7}$ brown block can be generated; These relationships can be specified by defining the number of blocks required for each creation and transformation. In the experimental design, the specific values used are provided. However, it is worth noting that non-sustainable short-term strategies become more advantageous when the condition $\alpha_{1}>\alpha_{3}$ is met, and the experimental design meets this criterion.

Moreover, with each turn, the biosphere is degraded in proportion to the players’ cumulative sum of non-renewable blocks. Specifically, for every black or red block owned by players in a turn, a brown block is consumed. An exception to this rule exists: for each player, each green block can sustain a red block, preventing the consumption of the shared brown blocks stock. For instance, if a player possesses eight green blocks and ten red blocks, only two brown blocks are consumed per turn for the upkeep of the red blocks. The rationale behind this rule is to offer agents the opportunity to compete using red blocks without necessarily depleting the shared biosphere, thereby incorporating a sustainable dimension to the competition.

The final rule pertains to the use of military capacity, represented by red blocks, which can be deployed against other players. A player may launch an attack once per turn. When two players engage in combat, the number of red blocks each player loses is determined by the smaller count of red blocks between the two opponents. If, at the end of the engagement, the attacking agent retains any red blocks, it appropriates production blocks from the defending player. Initially, it takes one black block for each red block that survived the aggression. If the count of remaining red blocks exceeds the black blocks owned by the defender, an equivalent number of green blocks is seized, up to the lesser of the remaining red blocks or the green blocks held by the defending player. If there are no any green blocks available, the defending agent is defeated.

##### Formal description

The formal description of the game depicts the dynamics of the players’ blocks for a single turn, not how they are supposed to make decisions, which is not part of the game’s rules. The following variables correspond to player *i*’s stock at the beginning of turn *t*.

(1) *i*, which is the player acting at turn *t.*
(2) $b_{t}^{i}$, $g_{t}^{i}$, and $r_{t}^{i}$: The quantities of black, green, and red blocks owned by player *i* at turn *t.*
(3) $e_{t}$: The number of brown blocks at the beginning of turn *t.*
(4) $bb_{t}^{i}$, $gb_{t}^{i}$, $rb_{t}^{i}$, $gg_{t}^{i}$, $bg_{t}^{i}$, $rg_{t}^{i}$, $eg_{t}^{i}$: Player *i* decisions regarding which resource will be utilized to produce another resource at turn *t.*
(5) *j*: The player being attacked by player *i.*
(6) $a_{t}^{i}$: A Boolean variable indicating whether the player *i* opts to attack at turn *t.*
(7) $b_{j,t}^{i}$, $g_{j,t}^{i}$, $r_{j,t}^{i}$: The quantities of black, green, and red blocks, respectively, owned by player *j* at the onset of turn *t.*
(8) $b_{\text{in},t}^{i}$ and $g_{\text{in},t}^{i}$: Variations in the number of blocks in player *i*’s inventory, relating to blocks subtracted from player *j* in a successful aggression.
(9) $r_{\text{out},t}^{i}$: The number of red blocks player *i* loses during an attack.
(10) $r_{s,t}^{i}$: The number of player *i*’s red blocks that endure the conflict.

The evolution of each stock is described by the following set of equations:

(Equation 1) $$b_{t+1}^{i}=b_{t}^{i}+\alpha_{1}bb_{t}^{i}+\alpha_{4}bg_{t}^{i}-gb_{t}^{i}+b_{\text{in},t}^{i}$$

(Equation 2) $$g_{t+1}^{i}=g_{t}^{i}+\alpha_{3}gg_{t}^{i}+\alpha_{2}gb_{t}^{i}-bg_{t}^{i}+g_{\text{in},t}^{i}$$

(Equation 3) $$r_{t+1}^{i}=r_{t}^{i}+\alpha_{5}rb_{t}^{i}+\alpha_{6}rg_{t}^{i}-r_{\text{out},t}^{i}$$

(Equation 4) $$e_{t+1}=e_{t}+\alpha_{7}eg_{t}^{i}-b_{t}^{i}-\max\left(r_{t}^{i}-g_{t}^{i},0\right)$$

In the model, there are seven distinct production constraints, each corresponding to a specific resource-production determination.

(Equation 5) $$bb_{t}^{i}\leq b_{t}^{i}-gb_{t}^{i}-rb_{t}^{i}$$

(Equation 6) $$gb_{t}^{i}\leq b_{t}^{i}-bb_{t}^{i}-rb_{t}^{i}$$

(Equation 7) $$rb_{t}^{i}\leq b_{t}^{i}-bb_{t}^{i}-gb_{t}^{i}$$

(Equation 8) $$gg_{t}^{i}\leq g_{t}^{i}-bg_{t}^{i}-rg_{t}^{i}-eg_{t}^{i}$$

(Equation 9) $$bg_{t}^{i}\leq g_{t}^{i}-gg_{t}^{i}-rg_{t}^{i}-eg_{t}^{i}$$

(Equation 10) $$rg_{t}^{i}\leq g_{t}^{i}-gg_{t}^{i}-bg_{t}^{i}-eg_{t}^{i}$$

(Equation 11) $$eg_{t}^{i}\leq g_{t}^{i}-gg_{t}^{i}-bg_{t}^{i}-rg_{t}^{i}$$

Subsequently, the outcomes of the confrontations are determined based on the aforementioned constraints and mathematical formulations.

(Equation 12) $$r_{\text{out},t}^{i}=a_{t}^{i}\;\min\left(r_{t}^{i},r_{j,t}^{i}\right)$$

(Equation 13) $$r_{s,t}^{i}=r_{t}^{i}-r_{\text{out},t}^{i}$$

(Equation 14) $$b_{\text{in},t}^{i}=a_{t}^{i}\;\min\left(r_{s,t}^{i},b_{j,t}^{i}\right)$$

(Equation 15) $$g_{\text{in},t}^{i}=a_{t}^{i}\;\min\left(\min\left(r_{s,t}^{i},b_{j,t}^{i}\right),g_{j,t}^{i}\right)$$

(Equation 16) $$b_{j,t+1}^{i}=b_{j,t}^{i}-b_{\text{in},t}^{i}$$

(Equation 17) $$g_{j,t+1}^{i}=g_{j,t}^{i}-g_{\text{in},t}^{i}$$

(Equation 18) $$r_{j,t+1}^{i}=\max\left(r_{j,t}^{i}-a_{t}^{i}r_{t}^{i},0\right)$$

#### Agent-based model

This section presents a simulation model of the sustainability game. For the code, please see the Code Availability section at the end of the paper. In the agent-based model, the primary focus is not the mere implementation of the rule sets into software, which is trivial, but the design of how players behave under different environmental conditions.^40^ The subsequent implementation by autonomous software objects, referred to as “agents”,^73^ is a direct result of this design activity.^74^ While the previous section describes the game’s rules, it does not detail how each agent makes decisions. Therefore, the dynamics of decision-making variables such as $bb_{t}^{i}$, $gb_{t}^{i}$, $rb_{t}^{i}$, $gg_{t}^{i}$, $bg_{t}^{i}$, $rg_{t}^{i}$, $eg_{t}^{i}$, $a_{t}^{i}$, and *j* are not specified.

**Figure 2:**
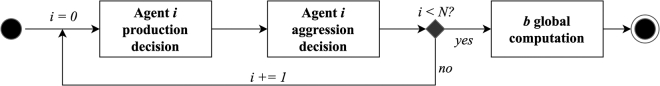
Flow diagram of model scheduling at each time-step *t*

In this model, there is a single species of agents: players. Each simulation begins with a set number of agents *N* and can last for *T* time-steps. At every time-step, each agent in the game can make a decision, resulting in agents making up to *T* decisions in every simulation. Figure 2 presents the scheduling for each time-step. The schedule comprises three main phases: production, aggression, and brown blocks computation.

Production is regulated by seven production preference states $pb_{x}^{i}$, in which agents exhibit heterogeneity. At every time-step *t*, an agent has a production capacity $C_{t}^{i}=g_{t}^{i}+k_{t}^{i}$. Therefore, the expected proportion of new blocks *x* produced by agent *i* at time *t* is given by $p{\left(x\right)}_{t}^{i}=C_{t}^{i}\frac{pb_{x}^{i}}{\sum pb_{y}^{i}}$. As a result, the larger the value of $pb_{x}^{i}$, the more likely the input production block $x_{k}$ will be used to generate the output block $x_{l}$.

In the decision-making process regarding whether to attack a specific connected agent, preferences $pa_{1}^{i}$, $pa_{2}^{i}$, and $pa_{3}^{i}$ are crucial. Specifically, the number of blocks held by potential target agents affects the likelihood of them being attacked, assuming the agent has decided to launch an attack. For instance, if agent *i* has a high value of $pa_{3}^{i}$, the preference to attack agent *j* increases with $r_{t}^{k}$.

This implementation, based on the combination of different $pb_{x}^{i}$ and $pa_{x}^{i}$, allows the agent to adopt potentially sophisticated strategies by combining various behavioral parameters.

The final phase of block computation involves two main tasks: determining the current count of brown blocks $e_{t}$ and ending the game if $e_{t}\leq 0$. The two preceding stages encompass the agents’ decision-making processes.

Agents’ decision-making is influenced not only by behavioral parameters but also by their risk preferences ^75^, which dictate how agents handle uncertainty in both production and aggression decisions. In each case, agent *i*’s risk preference is represented by a single risk sensitivity parameter $rs_{k}^{i}$ for the decision class *k* (either attacking or production). For a specific decision *k*, an agent is considered risk-averse when $rs_{k}^{i}>0$. On the other hand, an agent is deemed risk-seeking when $rs_{k}^{i}<0$. When $rs_{k}^{i}\approx 0$, agents display risk-neutral behavior. The parameters $rs_{r}^{i}$ and $rs_{a}^{i}$ define each agent *i* and cannot be adjusted during the simulation. Concerning both resource production and attack, agents face two risky decisions at each time-step *t*:

(1) Whether to produce a resource that consumes the livestock $e_{t}$, such as a black or a red block.
(2) Whether to attack one of the available agents.

Parameters $rs_{r}^{i}$ and $rs_{a}^{i}$, respectively, weigh the evaluation for the risky decision *d*, reflecting agent *i*’s preferences.

Moreover, $rs_{r}^{i}$ is also employed to modify agents’ behavior. It dictates how each agent adjusts its production preferences $pb_{x}^{i}$. Specifically, agents can make predictions about future states of the biosphere $b_{t}$. These predictions are formulated using a linear model, trained at every time-step *t* with the states of $b_{t}$ from the preceding $t_{m}$ time-steps, where $t_{m}$ represents the agent’s memory length. A prediction is then made, extrapolating the value of $b_{t+t_{l}}$, where $t_{l}$ is the forecast length. Both $t_{m}$ and $t_{l}$ are constant for all agents. Therefore, when the anticipated future biosphere stock $b_{t+t_{l}}<0$, agents begin to alter their production preferences $pb_{x}^{i}$ at a learning rate lr. The updating effect is positive (so that $s_{x}=1$) for $pb_{3}^{i}$, $pb_{4}^{i}$, and $pb_{7}^{i}$, but negative (i.e., $s_{x}=-1$)for $pb_{1}^{i}$ and $pb_{2}^{i}$. Thus, the more agent *i* anticipates potential biosphere exhaustion in the near future, the more it shifts toward a sustainable approach. Each behavioral parameters is updated by the following equation.

(Equation 19) $$pb_{x^{\prime }}^{i}=pb_{x}^{i}\cdot \left(1+\min\left(\frac{b_{t}}{b_{0}},1\right)\cdot rs_{r}^{i}\cdot s_{x}\right)$$

In this simulation model, agent interactions are governed by a single-level network.^76^ This network has a predetermined average number of connections $n_{c}$, reflecting the degree of interconnectedness among agents. This network structure is meant to force each agent to attack only neighboring agents. This limitation highlights the pivotal role the network’s structure has in shaping the system’s dynamics. Moreover, when agent *j* is eliminated, the attacking agent *i* inherits its neighbor set $N^{j}$, excluding those already connected to both agents. This inheritance mechanism is designed to simulate real-world scenarios where one state invades another and acquires its borders.

#### Model assumptions

The assumptions underlying our model can be methodically categorized into two distinct components: those pertinent to the game itself, and those associated with the ABM approach applied to the game. This division allows for a clearer understanding of the research’s foundations, distinguishing between the inherent rules and the behaviors of agents within the simulated game environment.

The game delineates a scenario in which players can adopt one of two overarching meta-strategies: competition or cooperation.^77^ Although this dichotomy is emblematic of traditional strategic formulations, our model introduces two additional dimensions along it. Firstly, there is a sustainability dimension, whereby the choice to compete directly impacts and potentially depletes the available natural resource stock, embodying the environmental consequences of strategic decisions. Secondly, the temporal dimension is considered, emphasizing that choices made in the present have enduring effects on future states. This approach elicits the tangled balance between immediate strategic gains and their long-term ecological effects. The underlying assumptions of the game are specifically tailored to address these key elements. We posit that decision-making entities, which in practical terms are nation-states, possess the autonomy to determine their production type and to perform military actions, having then the capacity to choose whether to engage in a military production escalation. This strategic choice is assumed to affect their survival prospects. It is important to note that our model intentionally excludes other potentially relevant dimensions, such as trade relationships or cultural exchanges. This exclusion is deliberate, serving the explicit purpose of concentrating our research on specific elements. Furthermore, the game incorporates additional, secondary assumptions. Firstly, it presupposes the feasibility of a wholly sustainable economy,^78^ suggesting that survival through exclusively green blocks is attainable. Secondly, the game acknowledges a detrimental interaction between military capabilities and renewable resources, positing that the augmentation of military capacity precipitates the depletion of these resources.

In contrast, the agent-based model posits that real-world entities’ behavior can be effectively simulated by explicitly encoding their attitudes toward various factors, such as uncertainty. So, although real-world entities may adopt risk-averse or risk-seeking behaviors through more complex mechanisms (such as the biological neural networks found in living organisms), the underlying assumption is that is feasible to approximate nations’ decision-making with specific parameters. Furthermore, the model presupposes that when agents make decisions based on estimations informed by their previous knowledge, they consistently employ a linear extrapolation. An additional assumption inherent to the model is the treatment of all national borders as uniform, represented by nondescript links without distinctive characteristics. Although this approach represents a simplification of reality, it is adopted to avoid the necessity of making further assumptions about the properties of these links, and facilitates a more streamlined analysis of network structure.

#### Experimental design

**Figure 3:**
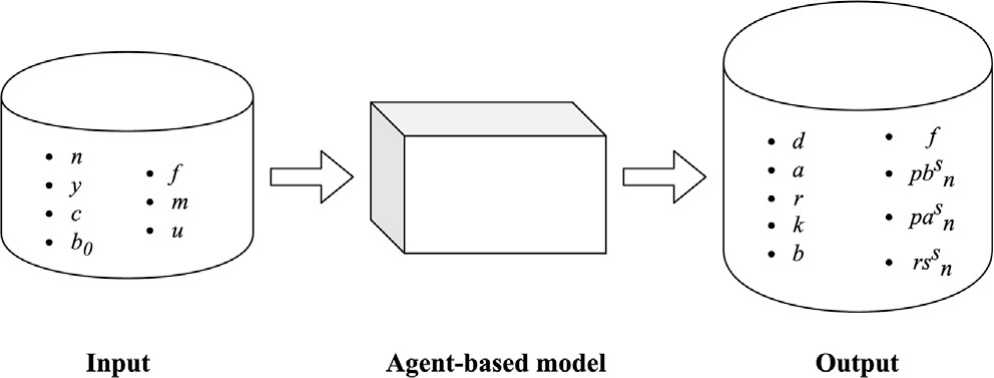
Black-box representation of the experimental setup

As declared in the introduction, the aim of this work is not to test a specific hypothesis, but to examine when it occurs in a game with different short-term and long-term incentives, and to observe the environmental conditions under which it is more likely or can be averted. Consequently, there were no strong initial assumptions about the values to assign to the parameters of interest.^79^ In situations where available computational power and model size permit, an extensive grid sampling exploration can be undertaken.^80^^,^^81^ This method involves running the model numerous times, drawing parameter values from random uniform distributions. In this case, sampling was carried out randomly. From a black-box perspective, this approach focuses on specific outcomes while ignoring the inner workings (see Figure 3). The model was simulated 100′000 times for 200 time-steps.

For each simulation run *r*, eight different parameters were randomly sampled from a continuous or discrete uniform distribution (see Table 3 for the sets of values each parameter could take). The parameters were the number of agents *n*, the network type *y*, the mean number of connections per node *c*, the stock of shared brown blocks at the initial time-step $t_{0}$ $b_{0}$, the forecasting length *f*, the forecasting memory *m*, and the preference update rate *u*.

**Table 3:** Parameters sampling for each simulation *r*

Parameters	Sampling set
*n*	n∈{10,11,…,199,200}
*y*	$y\in \left\{y_{1},y_{2},y_{3}\right\}$
*c*	c∈{2,3,…,19,20}
$b_{0}$	$b_{0}\in \left\{10000,10001,\ldots ,99999,100000\right\}$
*f*	f∈{1,2,…,19,20}
*m*	m∈{1,2,…,19,20}
*u*	u∈{0,0.0001,…,0.1999,0.2}

The number of agents *n* depicts how many agents are in the network at the beginning of the game $t_{0}$. We decided to study this parameter to observe if the scale of the system affects the results, as it can happen in behavioural-based agent-based models.^82^ The potential range extends for a single order of magnitude. Simulation results showed that the effect of a bigger population in the results stops much before our extreme value. For this reason, we considered the employed range reasonable. *y* denotes the network type. In this experiment, we examined three classic network configurations: Erdős-Rényi ($t_{1}$),^83^ Watts-Strogatz ($t_{2}$),^84^ and Barabási-Albert ($t_{3}$).^85^ The Watts-Strogatz model characterizes small-world networks. It begins with a regular lattice^86^ and introduces randomness via edge rewiring, yielding networks with high clustering and short average path lengths. Conversely, the Barabási-Albert model describes scale-free networks wherein nodes preferentially connect to well-established nodes, resulting in a power-law degree distribution^87^ with a few predominant hubs. The Erdős-Rényi model generates a random graph in which each node pair has an equal chance of connection, devoid of the pronounced clustering found in small-world networks or the scale-free degree distribution evident in the Barabási-Albert and Watts-Strogatz models. Such characteristics frequently appear in real-world networks.^88^ While this doesn’t encompass all potentially relevant network structures,^89^ these classic models, widely recognized in the literature, vary in their alignment with real-world social systems.^90^ We considered different network structure since the distance of connection between agentic entities both in simulated models^91^ and in real-world settings^92^ appears to have an effect on the overall evolution of the system. The number of connections per node *c* indicates the interconnectedness of the network. While the structure of the network dictates the distribution of connections among the *n* nodes, the total number of connections is given by nc. The level of connectivity can influence the final distribution of stocks and resources,^93^ which is why it is included in the analysis. $b_{0}$ represents the initial stock of brown blocks, the game’s common resource. Similar to *n*, the maximum amount was chosen so that its effect on the output was no longer observable. Empirical evidence indicates that the level of available resources influences the final state of a system in a sustainability context.^42^ The forecasting length *f* represents the number of future time-steps for which agents can perform linear extrapolation to decide whether to change production preferences. Given that each simulation lasts 200 time-steps, $\max\left(f_{r}\right)=20$ appears to be a reasonable value. Similar to *n* and $b_{0}$, results indicate that the effect of the parameters on the simulation’s observable outputs remains unchanged for values near the boundary of the selected sampling range. The forecasting memory *m* represents the number of past time-steps for which an agent gathers the values of $B_{t}$ to construct a linear model and predict the future status of the biosphere at time t+f. The value is chosen to mirror the forecasting length. Finally, *u* represents the preference update rate, indicating how quickly agents update their production preferences. max(u)=0.2 suggests that agents cannot modify their preferences by more than 20% at each time-step, so that in the fastest case it takes 5 time-steps to complete change its preferences. Previous studies indicate that the ability to learn, the speed of learning, and memory can all influence the evolutionary direction of a multi-agent system.^94^^,^^95^

The analysis of this parameters’ set could help determine the effects of various system features on the overall simulation results. More precisely, there are three distinct categories.

(1) Network interactions: this category encompasses parameters that influence how agents interact with each other. Parameters defining this category include *n*, *y*, and *c.*
(2) Resource availability: this category includes parameters that define the initial level of resources in the system. The sole parameter in this category is $b_{0}$, although the quantity of the initial biosphere should also be studied in relation to the number of players *n* utilizing it.
(3) Information processing capacity: this category encompasses parameters that influence agents’ ability to assimilate and respond to information. Parameters like *f* and *m* outline the temporal depth of agents’ predictive and retrospective insights, respectively. Furthermore, *u* determines how quickly agents can learn from environmental changes.

In a black-box representation of the agent-based model experimental setting, the outputs are observable variables, that is, the values used to observe the model’s behavior. In this experiment, for each simulation run *r*, three sets of variables are collected.

(1) Simulation state outputs: these define the characteristics of the simulation. The sole element in this set is the simulation duration *d.* Given the maximum length of the simulation *T*, d<T if the socio-ecological system simulated collapses or if there is a single winner.
(2) System state variables: these define the status of the simulated system at the end of the simulation. They include the number of agents at the end of the simulation run *a*, and the sum of the levels of each agent’s red blocks br, black blocks bk, green blocks bg, and brown blocks bb.
(3) Behavioral variables: these represent the mean of agents’ behavioral parameters collected from the agents. There are two types of behavioral parameters collected: those from agents who survive until the end of the simulation and those from agents defeated before the simulation’s end. Table 4 provides an overview of the variables belonging to this category.

**Table 4:** Behavioral outputs of each simulation *r* are collected both for agents that survive until the last time-step *d* (s=1) and for agents that were defeated during the course of the simulation (s=0)

Name	Description
$pb_{1,r}^{s}$	Mean preference to produce *k* using *k*
$pb_{2,r}^{s}$	Mean preference to produce *k* using *g*
$pb_{3,r}^{s}$	Mean preference to produce *g* using *k*
$pb_{4,r}^{s}$	Mean preference to produce *g* using *g*
$pb_{5,r}^{s}$	Mean preference to produce *r* blocks using *k*
$pb_{6,r}^{s}$	Mean preference to produce *r* blocks using *g*
$pb_{7,r}^{s}$	Mean preference to produce *b* blocks using *g*
$pa_{1,r}^{s}$	Mean preference to produce a player with *k*
$pa_{2,r}^{s}$	Mean preference to produce a player with *g*
$pa_{3,r}^{s}$	Mean preference to produce a player with *r*
$rs_{r,r}^{s}$	Mean risk sensitivity regarding resource production
$rs_{a,r}^{s}$	Mean risk sensitivity regarding attack

The agent-based model was implemented in Python 3.11.3 without using any specific framework for ABM. Random numbers in each simulation were generated with Numpy 1.24, using a given random seed that was recorded alongside the simulation results to ensure replicability. Data analysis and treatment were also conducted in Python 3.11.3.

## Results

This section exhibits the analysis of the data gathered from the exploration of the agent-based model of the sustainability game previously defined.

**Figure 4:**
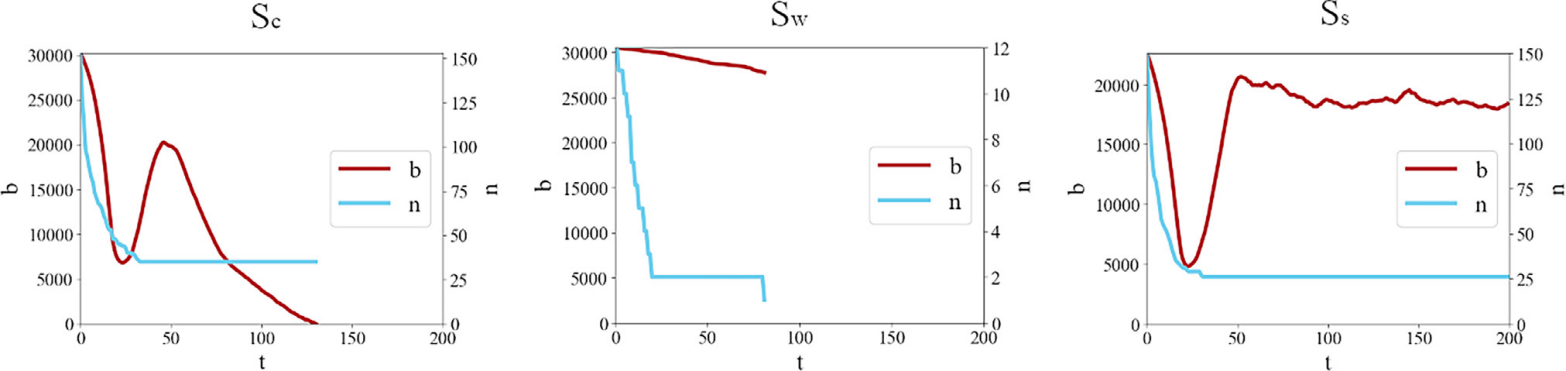
Time series of the number of brown blocks *b* and number of players *n* for three illustrative simulations, each belonging to a different scenario

From the analysis of the results, three possible scenarios *S* emerged. First, a set of simulations in which multiple agents survive until the final time-step *T*. We refer to this as the “surviving scenario” or $S_{s}$. Second, a scenario in which only one agent survives. This can only occur when all other agents are progressively defeated during the course of the simulation, and the simulation ends before *T*. We refer to this as the “winner scenario” or $S_{w}$. Lastly, there is a group of simulation runs in which the biosphere is completely consumed, such that at the last time-step d≤T, $b_{r,d}=0$. We term this the “collapse scenario” (or $S_{c}$), based on the assumption that a real-world society that depletes all of its biosphere would likely experience at least a societal collapse. Table 5 depicts the main statistics for $S_{s}$, $S_{w}$, and $S_{c}$, while Figure 4 presents an example of behavior for each scenario.

**Table 5:** Main simulation statistics per each scenario

Scenario	# sim	E[d]	E[a/n]
collapse	13832	41.69	0.175
single winner	1928	63.07	0.040
collective win	84240	200.00	0.145

E[d] is the mean duration of the simulation in a given scenario in time-steps, while E[a/n] is the mean fraction of agents survived until time *d*.

**Figure 5:**
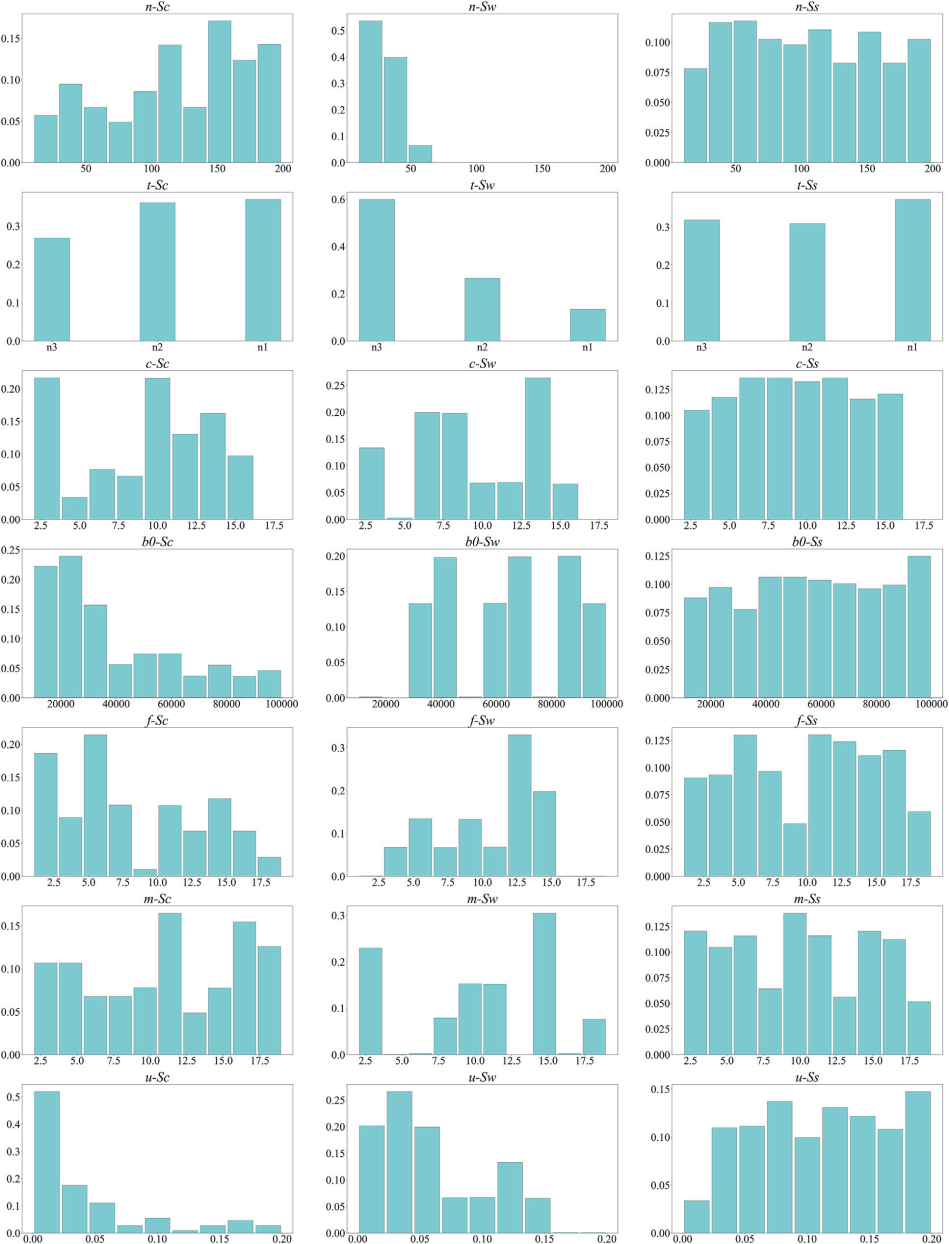
Frequency of parameter values for each simulation, categorized by parameter and scenario This table aims at highlighting the influence of parameters on the final scenario. Given that each variable has a uniform sampling probability, displaying the distribution of parameters for each scenario allows for an understanding of the impact of each parameter on the outcome.

The large number of simulations resulting in population survival can be attributed to the thorough exploration of the parameter space and its inherent structure. The variables most influencing the probability of collapse are $b_{0}$ and *u*, with Figure 5 illustrating an increased collapse risk as these values approach 0. Since, in the majority of the simulations, $b_{0}$ and *u* are far from the values that are likely to lead to $S_{c}$, a greater proportion of simulated populations endure without collapsing.

Table 6 presents the mean values of parameters for each final scenario: $S_{s}$, $S_{w}$, and $S_{c}$, along with their standard deviations. The presentation of results is designed to emphasize the cause-effect dynamics between environmental parameters, facilitating a clear understanding of the individual impact of each variable on the simulation’s outcome, regardless of the scenario *S*. Each variable outlined in Table 6 requires a discussion. On one hand, a thorough examination ensures a complete understanding of the model’s behavior. On the other hand, such a discussion is crucial for potentially extrapolating the model’s findings to real-world contexts, highlighting the broader implications and significance of this research, as well as its limitations.

**Table 6:** Mean values of each numerical explored parameters for the three scenarios

*p*	$E\left[p_{S_{s}}\right]$	$V\left[p_{S_{s}}\right]$	$E\left[p_{S_{w}}\right]$	$V\left[p_{S_{w}}\right]$	$E\left[p_{S_{c}}\right]$	$V\left[p_{S_{c}}\right]$
*c*	10.43	5.39	9.31	4.36	10.14	5.05
*n*	121.6	53.61	24.97	12.96	104.4	53.42
$b_{0}$	39186	24517	63600	22393	56869	25870
*f*	8.62	5.56	10.58	3.41	10.35	5.34
*m*	11.39	5.27	11.39	5.61	10.44	5.1
*u*	0.04	0.05	0.06	0.04	0.11	0.05

The network type, which is not a numerical parameter, is depicted exclusively in the histogram.

The number of agents *n* has significant implications for the dynamics observed in the simulations. In simulations with a low initial number of agents, the likelihood of a single entity emerging as the sole winner is higher. This result is intuitively understandable, given the significant time and effort needed to out-compete many rivals, and considering that resources could be depleted during this competitive process. With fewer agents in the environment, each has more freedom to focus on strategic attacks against its peers without the added pressure of multiple entities depleting the shared resource simultaneously. Essentially, these findings suggest that aggressive strategies are more effective in contexts with limited competition, even if they aren’t always the optimal strategy.

The type of network used in a simulation (second row of Figure Figure 5), represented by *y*, had a marked effect on the outcomes. Although the Erdos-Rényi networks influenced the probability of having multiple winners more, the difference was not substantial. In contrast, 60% of the simulations that ended with a single winner used the Barabasi-Albert network configuration, while only 10% employed the Erdos-Renyi setup. A potential reason for these outcomes lies in the intrinsic properties of the networks. Both small-world and scale-free networks facilitate efficient transitions between different regions of the network. This means agents have broader access to competitors. Specifically, small-world networks (such as those created with the Watts-Strogatz algorithm) have two main features: a higher clustering coefficient compared to regular lattices, and an average path length similar to that of random graphs. The clustering coefficient reflects nodes’ tendency to group, indicating local interconnectedness, whereas the average path length represents the average number of steps to connect any two nodes. Moreover, a network is “scale-free” if its degree distribution follows a power-law distribution. This means that while most nodes have few connections, a small subset, known as hubs, has exceptionally high connectivity. This uneven distribution significantly affects our simulations. The more interconnected a node is, the more likely it is for a dominant player to connect with a weaker player, potentially leading to the latter’s elimination. When considering this dynamic across all agents in a simulation *r*, a network structure that enables easy transitions tends to result in a single winner scenario. Furthermore, when other factors remain the same, scale-free networks, especially those created using the Barabasi-Albert algorithm, show a decreased likelihood of collapse. Scenarios that might lead to a collapse in other networks often result in individual victories with a Barabasi-Albert network structure.

The parameter *c*, which represents the number of connections within the network, does not seem to have a strong effect on the simulation outcomes. This observation is especially striking when contrasted with earlier findings about network structure. It highlights that the structural characteristics of a network have a more profound influence on the dynamics and potential collapse of a socio-ecological system than the mere number of connections in the network. This insight stresses the significance of structural configurations over simple connectivity density in determining system resilience.

The initial quantity of resources in the system $b_{0}$ unsurprisingly has a strong influence on the simulations’ final state. Specifically, the $S_{c}$ scenario becomes significantly more likely under conditions of scarce resources. As illustrated in Figure 5, collapse is not exclusive to low-resource scenarios but is decidedly more common. Furthermore, situations of individual dominance or sole victories are almost absent under resource-poor initial conditions. This observation underscores the insight that intense competition emerges as a dominant sustainable dynamic when the system has an ample reservoir of resources, allowing non-cooperative dynamics to persist longer.

**Figure 6:**
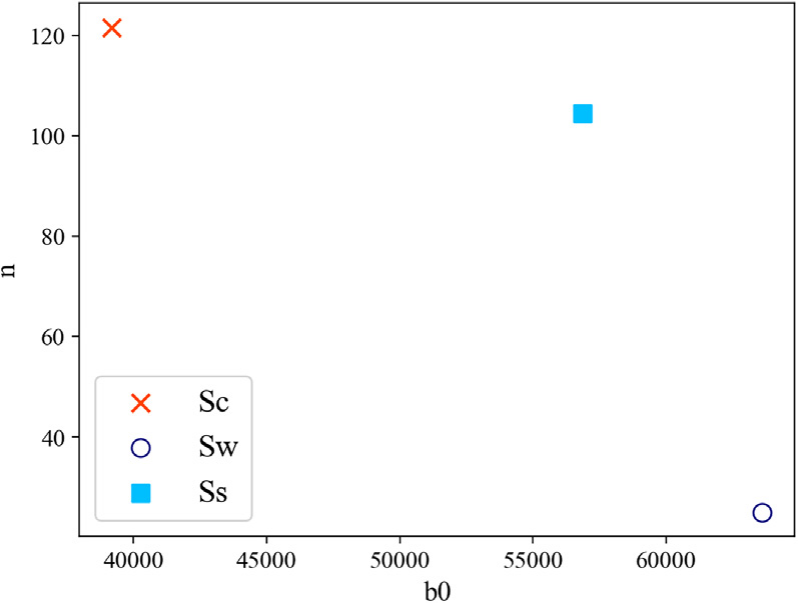
Scatterplot of E[n] and E[b_0] for each scenario *S*

A notable observation emerges when considering that the initial number of brown blocks is not the sole determinant of system resources, which are also influenced by agent interactions, as each agent can consume brown blocks. This interplay is vividly illustrated in Figure 6, where the mean values of the number of agents E[n] and brown blocks $E\left[b_{0}\right]$ are plotted across scenarios $S_{c}$, $S_{w}$, and $S_{s}$. The overarching trend is notable. On average, $S_{w}$ presents with an abundance of initial resources and a limited number of agents. Thus, it can be inferred with a degree of confidence that $S_{w}$ conditions predominantly arise when the per capita resource availability is high, allowing agents to compete until singular dominance emerges without causing a systemic collapse. By contrast, conditions predisposed to collapse tend to exhibit an inverse relationship: an above-average number of initial agents vying for a limited resource cache. It is evident that as per capita resources diminishes, the system’s propensity for collapse increases. The multi-winner scenario, represented by $S_{s}$, occupies an intermediate position. Although $S_{w}$ was initially hypothesized as a median scenario between $S_{c}$ and $S_{s}$, this analysis offers a nuanced perspective. $S_{w}$ is not an intermediate state. Instead, $S_{s}$ appears to represent an unstable equilibrium poised between two stable equilibria. As stated at the beginning of this section, a high share of simulations concludes in $S_{s}$, mostly due to the set of parameters explored. However, the likelihood of this scenario does not imply its stability. For example, consider the other two ending scenarios. When an agent remains alone, no reason for competition exists, and it can focus solely on surviving, with no possibility of return. Similarly, if the biosphere is totally consumed, there is no possibility to revert to a previous state. On the other hand, in the $S_{s}$ scenario, resources are still available to be totally consumed, and competition is ongoing. Figure 4 suggests that, as long as multiple agents are alive, there can be potential for collapse or singular victory. Hence, even if it is the most likely state, and even if under some configurations it appears sustainable, it cannot be considered a stable state.

The *f* parameter reveals a notable trend: systems populated by agents with shorter forecasting horizons are more susceptible to collapse. This finding aligns with intuitive reasoning. When a population of agents possesses a longer foresight, they inherently have the capacity to anticipate forthcoming challenges. Consequently, these agents are better positioned to implement timely interventions, bolstering the system’s resilience and reducing its vulnerability to potential pitfalls.

The memory span of agents *m* does not exhibit a pronounced impact on the simulation outcomes. The muted influence of this parameter can be attributed to the nature of the predictions implemented in the agents. Specifically, the phenomenon being forecast in critical moments (i.e., when the time series of the brown blocks level is approaching 0) exhibits a monotonically decreasing trend. Hence, irrespective of possessing a long or short memory span, agents can adeptly infer the trajectory, ensuring the resultant predictions remain consistently accurate across varying memory lengths.

*u* appears to be pivotal in mitigating the appearance of $S_{s}$. While certain populations may still face collapse despite rapid learning rates, the probability of system collapse decreases notably with *u*. This relationship is evident even in simulations resulting in a one-winner scenario. This, combined with the low mean time simulations with one winner last, suggests that they occur when agents are going tight to finish the common biostock, but instead of stopping, they try to kick out other agents to remove the competitive element. Time series observations confirm this hypothesis.

**Figure 7:**
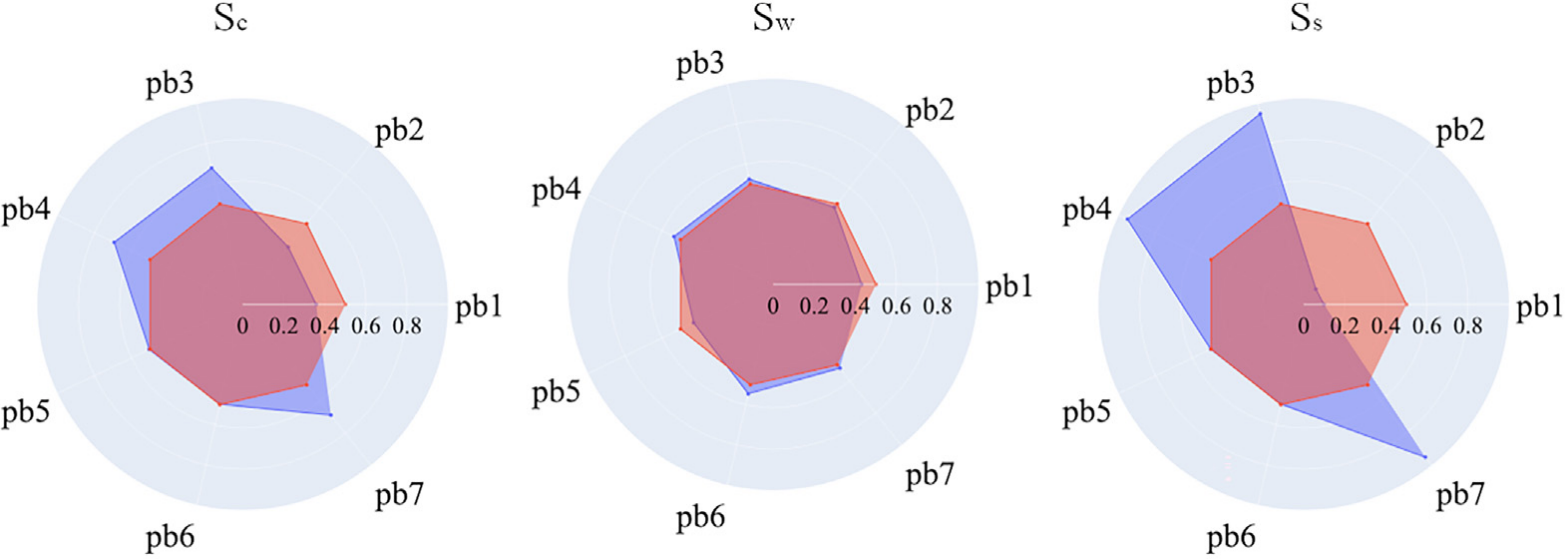
Mean of behavioral parameters per scenario The blue line depicts the situation and the beginning of each simulation *r*, where the expected mean of each behavioral parameter is 0.5. The red line depicts the mean effect of the adaptation process in each scenario.

In assessing the behavioral parameters of agents within the system (see Figure 7), a distinct direction in learning emerges based on the model design, in which agents can only learn to be more sustainable, not less. Even if learning follows a singular trajectory, its manifestation varies significantly across the three scenarios. In $S_{w}$, the disparity between the initial mean distribution of agents’ preferences and those of the surviving agents is subtle. This subdued difference can be attributed to the notably shorter mean simulation time, as indicated in Figure 4. Conversely, $S_{c}$ displays a tendency toward adaptation, albeit moderately. Here, while the process of adaptation begins, it often remains incomplete, as system collapse frequently occurs before full adaptation is achieved. In contrast, agent populations in the surviving scenario predominantly exhibit strong adaptation, underscoring its pivotal role in ensuring survival. This adaptive behavior predominantly results in three strategic choices: transitioning from non-renewable industrial capabilities to renewable ones, developing renewable industries using renewable capabilities, and rejuvenating the biosphere.

## Discussion

This work has several implications, both stemming from the significance of the topic and the results observed in the experiments.

The tension between addressing short-term concerns and long-term implications is relevant both regarding sustainability issues^96^ and geopolitical decision.^97^ Various examples elucidate this dichotomy,^98^ spotlighting immediate challenges juxtaposed against enduring sustainability issue.^99^^,^^100^ The commercial and industrial rivalries among states acts in a similar way,^101^ with governments in need of face immediate challenges even when long-term issues become clear.^102^

The results, which are indeed affected by the model design, do not suggest that mere avoidance of conflicts is a potential way out from the risk of collapse, since it does not guarantee survival. Instead, in this setting, agents are able to survive as a group when they embody competition in their sustainable behavior. The heightened awareness of existential risks ensures that while competition remains, it becomes a secondary priority. Essentially, more immediate existential threats take precedence in decision-making.

The way in which an existential threat is perceived as pressing depends on environmental perception and the capability to embody this perception in agents’ behavior. Thus, how acting components of a socio-ecological system gather and process information greatly influences its survival prospects. Specifically, the ability to forecast effectively allows simulated agents to adapt their behavior toward more sustainable practices. As a species, we have been aware since the early seventies that we might face sustainability issues that could lead to societal collapse, as scientists began highlighting the threat of resource exhaustion. However, as we now approach the ability to predict potential systemic collapses or extinctions, a significant challenge emerges: our willingness to modify our preferences. While data interpretation may be the initial phase of information processing, the subsequent and equally vital phase involves learning and adapting based on that data. Our findings suggest that within a framework of both competition and cooperation, populations exhibiting a slower rate of preference adjustment (i.e., they are less adaptive) are at an increased risk of collapse.

The interactions among nations and entities in competitive environments have historically played a pivotal role in shaping their ultimate standings in resource distribution. However, a novel insight emerging from our research is that the essence of these interactions may not hinge solely on the degree of interconnectedness, but more critically on the underlying structure of the network. This revelation underscores the need for deeper exploration to discern whether this phenomenon is exclusive to the settings examined in our study. While our initial assessment suggests broader applicability, rigorous investigations are necessary to determine if similar dynamics manifest in analogous contexts.

In this paper, we showed that the current configuration of our living conditions represents an unstable equilibrium, which has implications for managing the world as a socio-ecological system. On one hand, perturbations in the global situation, such as an increase in a given parameter or a switch in the nations interaction network structure, as observed in the nineties, could potentially shift the equilibrium in a specific direction. On the other hand, it’s crucial to recognize that in the real world, while we may not precisely know our position, limited efforts have been made to change our behavior. Conversely, many studies indicate that various biosphere level indicators are consistently declining. As a society, we might be in a situation where we are not close enough to perceived dangers to significantly alter our behavior. In this context, outcomes will depend not only on our behavior but also on technological adaptation, because in our model, we considered these production relationships between blocks to be stable.

For practitioners, the utility of research findings is enhanced through the inclusion of practical examples that illustrate key takeaways. First, governments could implement policies that encourage or mandate the use of predictive analytics in specific areas, such as resources or energy management systems. Such policies might prioritize investments in renewable energy and resources, which are influenced by long-term weather and climate forecasts. This action aligns with the simulation’s suggestion that enhanced forecasting abilities can mitigate the perception of temporal abundance and promote sustainable behavior. Second, planners could utilize agent-based simulation, similar to our simulation, to manage shared resources in specific areas, and to experiment the effects of different relationship structures and individual preferences on the overall system. Third, educational programs on sustainability should include predictive modeling of resource availability and consumption trends. By integrating these models into educational curricula, students and communities can better understand the importance of sustainable practices and their long-term benefits. This approach directly responds to the simulation’s findings on the importance of rapid behavioral adaptation among entities. Fourth, another actionable policy is the development of regulatory frameworks that require corporations to include sustainability forecasts in their annual reports, making them more accountable for their environmental footprint, and encouraging a shift toward a long-term perspective. Finally, sectors using shared resources, such as agriculture with water, can employ and improve data analytics and predictive models to optimize their use, without relying on the individual preferences of individuals. This approach is in line with the results’ emphasis on management of shared resources to prevent depletion.

### Limitations of the study

This paper identifies three primary limitations: stylization, linearity in prediction, and biosphere modeling. It is important to emphasize these to delineate the scope of the results, particularly given the significance of the topic.

The game is a stylized model of a real-world scenario. Therefore, it is essential to approach its results with caution and not accept them uncritically. We developed such models because, despite their simplifications, they are significant in providing an effective methodology to formulate strategies for setting non-testable *in vivo*, and also observe emergent phenomena in a controlled environment, free from statistical noises and ethical concerns. Instead of treating the outcomes as strategies to be applied universally to every socio-ecological model facing this specific short-term to long-term sustainability issue, we advise viewing them as foundational inspirations that can catalyze further investigations and analyses in subsequent studies. To ensure the results’ applicability to real-world scenarios, cross-validation through behavioral experiments or existing behavioral data are crucial. Such empirical verification could also offer a stronger foundation, bridging the divide between theoretical predictions and practical implementation.

Individuals often exhibit a tendency to make predictions linearly.^103^ However, this behavior is not uniformly observed across all countries^104^ and is not always applicable to organizations, such as governmental bodies. While assuming such linearity can be considered a realistic premise in specific contexts, it inherently limits the generalizability of our findings. Consequently, this poses a limitation on the broader applicability of the results, necessitating careful contextual interpretation.

In our study, we simplify the representation of the biosphere by treating it as a single, shared stock subject to renewal through industrial capacity. While this assumption provides a streamlined approach for our model, it abstracts significantly from the complexities of the real world. Specifically, our model overlooks two essential attributes of the biosphere: heterogeneity and partial renewability. The former pertains to the varied consumption patterns of individual countries that target specific portions of the biosphere, so that some areas could be more damaged than other. The latter refers to the inherent limitations in the possibility to regenerate resources: for example, once a species becomes extinct, it cannot be brought back, and similarly, depleted wood or fish reserves cannot be replenished only by employing renewable industrial capability.

Finally, in the construction of our model, we consciously opted not to account for technological changes, despite the evident existing rate of innovation observed in present times. This might seem an overly simplistic assumption given the complexities of real-world scenarios. However, our rationale was to cleanly isolate the impact of environmental parameters without the confounding influence of fluctuating technological advancements. While the past three decades have indeed witnessed strides in sustainability technologies, it’s worth noting that no truly disruptive breakthroughs have emerged within this domain.

### Conclusions

In this paper we have expanded on a prior sustainability game,^10^ wherein blocks idealize non-renewable and renewable resources, military capacity, and biosphere availability. Each agent can choose to invest in different stocks, aiming to survive as long as possible. Here, we have generalized the game and analyzed its outcomes on networks. The three possible outcomes we have seen occurring in the simulation are collapse, a single winner or a collective win. While the number of connections in the network do not have a marked effect on the outcome, the topology does: 60% of the simulations that ended with a single winner used the Barabasi-Albert scale-free network configuration, while only 10% employed the Erdos-Renyi topology. A more highly connected node means it competes with weaker players, thus increasing its likelihood of success. This echoes prior game theoretic findings on core-periphery networks.^105^

The probability of occurrences of each scenario also depends on other factors, such as the number of entities that are competing and consuming the resources, as well as the overall level of initial resources available. Results show that the multi-win scenario is the most likely, but they suggest it is also the only unstable one, with collapse or a single-win being more representative of the final steady states. Finally, information processing appears to play a role in how a population of agents can avoid collapse. More precisely, the more and the faster information can be processed, the more likely it is that collapse can be avoided.

The results suggest possible actions for sustainability practitioners dealing with a similar setting. Small-world network topologies of interactions are not recommended, while long-term forecasting should be encouraged. Also, if possible, the numerosity of entities competing within a non-cooperative framework should be reduced. Finally, the research outcomes emphasize the need for education and cultural dissemination for rapid behavioral change.

Also, the research outcomes generate a whole new set of questions to be successively tested by further empirical studies. First, the evaluation of how different strategies in managing shared resources are affected by different interactions’ structures. Second, whenever real-world entities (e.g., communities, nations) adapt their resource consumption and production behaviors in response to imminent resource scarcities. Third, assessing the influence of availability and processing of information regarding resource statuses, and relative future projections, on decision-making. Finally, identify how education and public awareness initiatives influence individual and collective decision-making toward more sustainable practices and what communication models have been most effective in changing the behaviors of a collective entity.

Finally, future developments for this research could include various enhancements to the model. Firstly, a version in which agents possess the capacity to “de-learn” sustainable behaviors might provide insights into the regression of sustainability initiatives. Another perspective worth exploring is the influence of the technological setting. By studying this, we can potentially discern which directions of investment are more relevant for increasing the chances of averting system collapse. Furthermore, translating the model into a game format with human players could be valuable. Such an implementation would enable us to verify whether the conclusions derived from the current simulations also hold true in more human-centric scenarios. Lastly, the game foundational to the model holds potential as an educational instrument designed to enlighten the public and stimulate policy discussions. Also, beyond its pedagogical objectives, this application could enable human participants to engage with the game directly, allowing for the documentation of their strategic choices and outcomes. Such player-generated data can then be compared with results and strategies derived from the model itself. This comparative analysis not only enhances understanding of the model’s dynamics but also offers valuable insights into human decision-making processes in similar scenarios, bridging theoretical predictions with practical human behavior.

## STAR★Methods

### Key resources table

**Table:** 

REAGENT or RESOURCE	SOURCE	IDENTIFIER
**Deposited data**		

Result data	https://osf.io/b7jty/?view_only=ef5b75ed47544d7c9793866016713684	https://doi.org/10.17605/OSF.IO/B7JTY

**Software and algorithms**		

Simulation model	https://osf.io/b7jty/?view_only=ef5b75ed47544d7c9793866016713684	https://doi.org/10.17605/OSF.IO/B7JTY

### Resource availability

#### Lead contact

Further information and requests for resources and reagents should be directed to and will be fulfilled by the lead contact, Francesco Bertolotti (fbertolotti@liuc.it).

#### Materials availability

This study did not rely or generate material.

#### Data and code availability

The simulation model, the data generated from the simulation, and the code used to analyze it, on which the results of this study rely, have been deposited to the following Open Science Framework repository: https://osf.io/b7jty/?view_only=ef5b75ed47544d7c9793866016713684. The DOI is listed in the key resources table. The repository contains all the code employed during the work. Any additional information required to reanalyze the data reported in this paper is available from the lead contact upon request.

### Method details

The research employs an agent-based model (ABM) to simulate a game where players make strategic decisions balancing short-term production and military goals against long-term sustainability. The game rules are expressed both qualitatively and mathematically to ensure clarity and replicability. Players interact within a predefined system of rules governing resource production, combat, and sustainability impacts, captured in detailed mathematical equations and decision-making processes.

Simulations were performed 100,000 times over 200 time steps, with a detailed sampling of parameters including agent count, network type, and resource stock levels. These parameters were drawn from uniform distributions to ensure broad exploration of potential scenarios.

The ABM posits that the simulated agents representing decision-making entities, such as nation-states, are autonomous in their production and military decisions, which affect their survival and impact on sustainability. The model abstracts real-world complexities, such as trade and cultural interactions, to focus on environmental and strategic dynamics.

### Quantification and statistical analysis

All statistical analyses were conducted using Python 3.11.3 on a 2023 MacBook Pro M2 Pro with 32GB of RAM. Data manipulation was facilitated by Numpy 1.24 and Pandas 2.0, while visualisations were generated through Matplotlib 3.7.2. The raw data was compiled from multiple CSV files located in a designated data folder, processed, and analysed to assess various metrics related to the study’s objectives.

The exact value of each relevant variable can be found in the figure legends, figures, and results section. The model was simulated 100000 times, generating 100000 observations contributing to each data point. For each simulation, outcomes such as survival rates and attack success were analysed (details are listed in the paper, and present in the available data). Sample size estimation was based on preliminary data to achieve sufficient power to detect differences between groups.

The mean and standard deviation were primarily used to describe central tendency and dispersion. Data visualization included bar graphs and scatter polar plots, providing a clear representation of the trends and variations within the data.
